# Accidental salinomycin intoxication in European fallow deer (*Dama dama* L.)

**DOI:** 10.17221/100/2023-VETMED

**Published:** 2024-01-23

**Authors:** Martin Svoboda, Oto Huml, Petr Chomat, Alena Honzlova, Josef Illek, Zdenka Svobodova, Lada Hofmannova, Helena Modra

**Affiliations:** ^1^Ruminant and Swine Clinic, University of Veterinary Sciences Brno, Brno, Czech Republic; ^2^Vedilab Ltd Plzeň, Plzeň, Czech Republic; ^3^Veterinary Clinic Dobrošovice, Sedlčany, Czech Republic; ^4^State Veterinary Institute Jihlava, Jihlava, Czech Republic; ^5^Large Animal Clinical Laboratory, University of Veterinary Sciences Brno, Brno, Czech Republic; ^6^Department of Animal Protection and Welfare and Veterinary Public Health, University of Veterinary Sciences Brno, Brno, Czech Republic; ^7^State Veterinary Institute Prague, Prague, Czech Republic; ^8^Institute of Environmental Science and Natural Resources, Mendel University in Brno, Brno, Czech Republic

**Keywords:** deer farm, creatin kinase, hyperkalaemia, hypochloraemia, urea

## Abstract

Salinomycin, belonging to ionophore antibiotics, has been used as a feed additive for poultry for its coccidiostatic effect. Poisoning by ionophore antibiotics has been reported in cattle and other sensitive animals due to the replacement of medicated feed and/or accidental overdoses. The aim of this paper is to report the toxicity of salinomycin for fallow deer and to describe the different levels of sensitivity of cervids to this substance. In the presented case study, a medicated feed containing ivermectin used for deworming red deer and fallow deer was accidentally contaminated with sodium salinomycinate in a concentration of 252.6 mg/kg. The contaminated feed was consumed by the animals over a period of four days. The mortality of fallow deer within 12 days was 58%. No mortality was recorded in the red deer. In the affected animals, clinical signs associated with acute and congestive heart failure were observed. The biochemical examination indicated prerenal azotaemia caused by circulatory insufficiency and ion imbalance. The histological examination revealed pronounced focal acute cardiomyopathy and massive subacute myopathy in the skeletal muscles.

The growing interest in deer farming and the increase in the fallow deer population in Europe have been recorded since the early 1980s (Bijl and Csanyi 2022). In 2016, the total number of farms had reached 817 in the Czech Republic with two-thirds of them being fallow deer farms and one-third of them being red deer farms, keeping around 18 700 animals in total (https://www.fedfa.com/fedfa-members/). This farming not only offers a business supplying deer meat, but also meat of higher quality than that of deer shot in the wild. However, the high density of animals in the farms favours a high prevalence and intensity of infections and losses in production. The prevalence of infections in fallow deer with gastrointestinal nematodes and lungworms in farm animals is 20–60%, therefore a reduction in the parasite infestation by chemotherapeutics is necessary for healthcare management (Borkovcova et al. 2013; Pilarczyk et al. 2015). Medicated feed is a useful and often used form of metaphylaxis for parasite infections in cervid farming systems although the use of medicated feed containing antimicrobials for metaphylaxis should only be allowed when the risk of an infection or infectious disease spread is high (Regulation EC No. 6/2019).

Feed business operators frequently manufacture a broad range of medicated feeds for different target animals containing different types of compounds, such as feed additives and/or veterinary medicinal products. Manufacturing different types of feed after each other in the same production line may result in "cross-contamination" and increase the risk of accidental replacement of pre-mixes or active substances (Dorne et al. 2013).

The aims of this article are to highlight the risk of feed additives and veterinary drug replacement in the manufacture of medicated feed, to describe the toxicity of salinomycin in feed in fallow deer, and to point out the diverse sensitivities in cervids.

## Case description

A case of poisoning was reported on a deer farm in the Czech Republic in January 2023. The farmer breeds a total number of eight red deer (*Cervus elaphus* L.) and 45 fallow deer (*Dama dama* L.).

A feed medicated with ivermectin with a declared concentration of 15 mg/kg was placed in the feeders in the amount of 40 kg (20 kg in the morning and 20 kg in the afternoon, divided among several places) for the purpose of deworming the game. The second day after the use of this feed, it was found that not all the feed had been eaten. According to the manufacturer’s instructions, another 40 kg was added in the same way. This feed was gradually consumed by the animals during the next 4 days. Approximately 10 kg of unconsumed medicated feed was then taken away.

Three fallow deer died 4 days after being fed with the medicated feed and another 22 fallow deer died over the next 3 days. The last victim was a fawn fallow deer euthanised 12 days after feeding with the medicated feed. The overall mortality in the fallow deer was 58%. Neither deaths nor clinical signs were recorded in the red deer.

The observed clinical signs in the fallow deer included a decrease in feed intake and anorexia and, later, shortness of breath and dyspnoea were added. Ataxia was observed approximately 30 min before death, and the affected animals were lying on their sides without signs of convulsions.

The investigation and consequential toxicological analyses revealed that the medicated feed contained ivermectin, but also a high concentration of salinomycin.

## MATERIAL AND METHODS

The following analyses conducted in the laboratories mentioned in brackets were made during the investigation: gross necropsy and histopathological analyses, bacteriological analysis, parasitological examination, rumen fluid examination, biochemical and haematological analyses of the blood (Vedilab Analytica Ltd., Plzeň, Czech Republic), virological analysis (Veterinary Research Institute in Brno), determination of ivermectin in the blood (State Veterinary Institute in Prague), and an analysis of the ionophore antibiotics (decoquinate, diclazuril, halofuginone, lasalocid, maduramicin, monensin, narazin, nicarbazin, robenidine, salinomycin, semduramicin) and ivermectin in the feed (State Veterinary Institute in Jihlava).

A gross necropsy, histological examination, and biochemical and haematological analyses were performed on an adult female who died on the 6^th^ day and the young animal euthanised on the 12^th^ day after the beginning of the exposure. Virological, bacteriological, parasitological, and rumen fluid examinations were only performed in an adult fallow deer which died on the 6^th^ day after the exposure.

### Histopathological analyses

Tissues were fixed in 10% buffered formalin, hydrated in isopropanol, placed in paraffin wax and cut at 4 μm. The incisions obtained were stained with haematoxylin-eosin and examined using light microscopy (magnification × 10–40).

### Virological examination

For the virological examination of the intestinal contents, transmission electron microscopy and negative staining method were used.

An approximate 20% suspension in a buffered physiological solution was prepared for the sample, which was then centrifuged at 5 000 *g* for 10 minutes. The supernatant was withdrawn. For the negative staining, the supernatant was suspended within a drop of distilled water.

The resulting suspension was covered with a grid-coated Formvar film (Sigma-Aldrich, Prague, Czech Republic) and carbon (Agar Scientific, Biedermannsdorf, Austria). The grid was removed from the suspension after 2 min, and the residual water was dried with a strip of filter paper. After drying, 2% of ammonium molybdate (Serva, Heidelberg, Germany) was placed onto the grid and the excess was dried for a few seconds, then the excess stain was dried with filter paper. The grid prepared in this way was observed under a Philips 208 S Morgagni electron microscope (FEI, Brno, Czech Republic) at 14 000–36 000 × magnification and an accelerating voltage of 80 kV.

### Bacteriological examination

The bacteriological examination of the intestine was carried out by routine cultivation on blood agar in an anaerobic and aerobic environment for 24 h at 37 °C. Subsequently, the grown cultures were isolated and identified.

The examination for *Salmonella* sp. was carried out by multiplying the faecal sample in Rappaport medium after 24 h of incubation at 42 °C in an aerobic atmosphere and re-inoculation on the selective Xylose-Lysine-Deoxycholate (XLD) medium (incubation for 24 h at 37 °C).

The targeted examination for *Clostridium perfringens* was performed during the primary culture on blood agar using the reverse Christie–Atkins–Munch-Peterson (CAMP) test.

At the same time, cultivation on End’s medium was performed in order to identify the lactose-negative bacteria and Gram staining of the stool sample for the possible detection of *Campylobacter* sp. bacteria.

The bacteriological examination of the liver and kidneys was carried out by a routine cultivation on blood agar in an aerobic environment for 24 h at 37 °C. Subsequently, the grown cultures were isolated and identified.

### Parasitological and rumen fluid examinations

The parasitological examination of the intestinal contents was performed by flotation using Sheather’s solution. TitroLine Easy titrator (VELP Scientifica Srl, Usmate, Italy) was used for the rumen fluid pH determination. The number of funnels was determined microscopically in a Fuchs-Rosenthal chamber at a × 10 magnification.

### Biochemical and haematological analyses

The biochemical parameters of the blood serum included the total protein (TP), albumin (ALB), globulin (GL), urea, creatinine, aspartate aminotransferase (AST), gamma-glutamyltransferase (GMT), creatin kinase (CK), calcium (Ca), phosphorus (P), magnesium (Mg), sodium (Na), potassium (K), chloride (Cl), and selenium (Se) were analysed using a Mindray BS-200 (Mindray Ltd., Huntingdon, Cambridgeshire, UK) analyser.

The following haematological indices were determined using a Mindray BC 30 vet haematology analyser (Mindray Ltd., Huntingdon, Cambridgeshire, UK): the number of red blood cells (RBCs) and white blood cells (WBCs), haemoglobin concentration (Hb), packed cell volume (PCV), mean corpuscular volume (MCV), mean corpuscular haemoglobin (MCH) and mean corpuscular haemoglobin concentration (MCHC). Blood smears, for the determination of the differential leukocyte count, were stained by May-Grünwald and Giemsa-Romanowski.

### Determination of ivermectin in serum

The ivermectin concentration in the serum was analysed by high-performance liquid chromatography (HPLC; Dionex UltiMate 3000, Dionex, Sunnyvale, California, USA) with MS/MS mass detection (AB Sciex 5500 QTRAP, Applied Biosystems, Waltham, Massachusetts, USA). Samples were extracted with acetonitrile (Chromasolv for LC-MS, Honeywell, Charlotte, North Carolina, USA), centrifuged at 850 g at 5 °C for 10 min in a Sigma 3-16K centrifuge (Sigma Laborzentrifugen GmbH, Osterode am Harz, Germany), and filtrated through a 0.45 μm nylon microfilter using a FilterBio^®^ NY Syringe Filter (Nantong FilterBio Membrane Co., Ltd, Nantong City, P.R. China).

The chromatographic analysis was performed on a Symmetry C18 column (150 × 3.0 mm, 3.5 μm; Waters Corporation, Milford, MA, USA) under the following conditions: mobile phase A (0.2% formic acid; LC-MS quality, Merck KGaA, Darmstadt, Germany) in acetonitrile (Chromasolv for LC-MS, Honeywell, Charlotte, North Carolina, USA), mobile phase B (5 mmol/l ammonium formate, puriss; Honeywell, Charlotte, North Carolina, USA) in 0.2% formic acid (LC-MS quality; Merck KGaA, Darmstadt, Germany), water solution (redistilled water Milli-Q plus, 5.5.10-8 Scm-1, Merck KGaA, Darmstadt, Germany; 85/15), and a flow rate of 0.4 ml/min, an injection volume of 30 μl, and a column temperature of 35 °C. Mass spectrometry detection: ionisation mode – electrospray ionisation (ESI) positive, an ion source temperature of 300 °C, an ion spray voltage of 5 500 V, a curtain gas of 25 psi, a collision gas medium, an ion source gas of 50 psi, with the scanning mode: MRM (892.4; 569.2; 307.0). The limit of quantification was 2.5 μg/kg.

### Determination of the ivermectin and salinomycin in the feed

The ivermectin in the feed was detected by HPLC with diode array detection (Waters Corporation, Milford, MA, USA) after ultrasonic extraction in a Bandelin SONOREX RK 510 ultrasonic bath (BANDELIN electronic GmbH & Co. KG, Berlin, Germany) with a solution of acetonitrile (Chromasolv gradient grade for HPLC; Honeywell, Charlotte, North Carolina, USA), methanol (Chromasolv for HPLC; Honeywell, Charlotte, North Carolina, USA) and redistilled water (Milli-Q plus, 5.5.10-8 Scm-1, Merck KGaA, Darmstadt, Germany; 45 + 45 + 10), after centrifugation at 810 g at 5 °C for 10 min in a Sigma 3-16K centrifuge (Sigma Laborzentrifugen GmbH, Osterode am Harz, Germany), and filtration through the 0.45 μm nylon microfilter using a FilterBio^®^ NY Syringe Filter (Nantong FilterBio Membrane Co., Ltd, Nantong City, P.R. China).

The chromatographic analysis was performed on an Xterra RP8 column (250 mm × 4.6 mm, 5 μm; Waters Corporation, Milford, MA, USA) under the following conditions: gradient elution with mobile phase A [acetonitrile – Chromasolv gradient grade for HPLC (Honeywell, Charlotte, North Carolina, USA) + methanol – Chromasolv for HPLC (Honeywell, Charlotte, North Carolina, USA)] + redistilled water [Milli-Q plus, 5.5.10-8 Scm-1 (Merck KGaA, Darmstadt, Germany), 45 + 45 + 10], mobile phase B [redistilled water – Milli-Q plus, 5.5.10-8 Scm-1 (Merck KGaA, Darmstadt, Germany), 60/40; 100/0], a flow rate of 0.5 ml/min, an injection volume of 10 μl, UV detection, a wavelength of a 246 nm, column temperature of 29 °C. The limit of quantification was set to 1.5 mg/kg; the repeatability of this method was 10% and the recovery was 70–91%.

Sodium salinomycin was determined after extraction with a solution of *n*-hexane (p.a., Lach-Ner, Ltd., Neratovice, Czech Republic) and ethyl acetate (puriss; Honeywell, Charlotte, North Carolina, USA) at a ratio of 1 : 4, and purification on a Silica solid phase extraction column, 500 mg (Waters Corporation, Milford, MA, USA) by HPLC with diode array detection after post-column derivatisation (Waters Corporation, Milford, MA, USA).

The chromatographic analysis was performed on a Nova-Pak C18 column (250 mm × 4.6 mm, 4 μm) (Waters Corporation, Milford, MA, USA), mobile phase methanol (Chromasolv for HPLC; Honeywell, Charlotte, North Carolina, USA) + 5% solution of acetic acid (Lach-Ner, Ltd., Neratovice, Czech Republic; 925 + 75), a flow rate of 0.8 ml/min, an injection volume of 50 μl, detection at a wavelength of 598 nm, a column temperature of 25 °C. The post-column derivatisation was performed in a Waters reaction module (Waters Corporation, Milford, MA, USA), with a reaction capillary length of 4 m, and a reactor temperature of 90 °C. The limit of quantification was set to 10 mg/kg; the repeatability of this method was 5% and the recovery was 80–99%.

## RESULTS

The gross necropsy performed on the fallow deer (adult female of average nutritional status) that died six days after the initiation of the contaminated feed consumption revealed congestion of the meninges, lungs, and several loops of the small intestine. The heart was of a normal size and shape; the valves were without deformations and inflammatory changes. Large vessels had normal diameters without any signs of morphological deviations. The myocardium and skeletal muscles were without macroscopic changes.

The necropsy of the young fallow deer euthanised 12 days after the beginning of the exposure showed slightly congested lungs, normal size and shape of the heart without deformations of the valves and any inflammatory changes, large vessels without any signs of morphological deviations from normal diameter, and myocardium and skeletal muscles without any macroscopic changes. The animal was unable to move, partially perceiving the surroundings and resisting handling.

The histopathological examination (death on the sixth day) showed an injection of vessels of the leptomeninges of the cerebrum, and oedema. There was dystrophy of the cerebellar neuron groups. Mild hyperaemia of the mucosa and infiltration of the villous stroma by lymphocytes and eosinophils were observed in the wall of the small intestine. The presence of numerous sarcocysts without inflammatory reaction was found in the myocardium.

The histopathological examination of the fallow deer euthanised 12 days after the initiation of the exposure showed a non-inflammatory and slightly thickened epicardium with significant hydropic degeneration of the subepicardial cardiomyocytes. In the myocardium, there were marked foci of hydropic degeneration of muscle bundles of cardiomyocytes with their swelling, loss of colourability and internal structure. The presence of a small number of small thin-walled sarcocysts filled with basophilic zoites was also recorded. Endocardial oedema with hydropic degeneration of adjacent cardiomyocytes was pronounced. Inflammatory-cellular infiltration was not detected.

**Figure 1:**
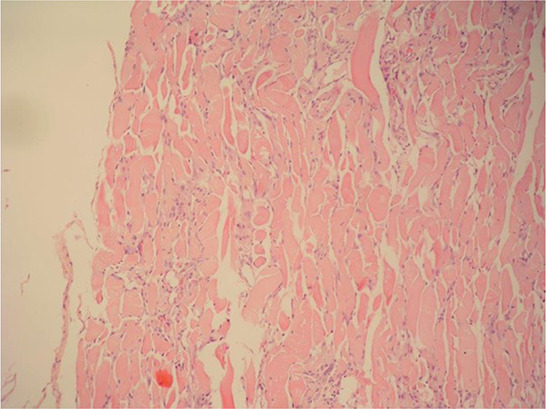
Pronounced skeletal muscle myopathy – disintegration and uneven strength of muscle fibres, their focal disintegration and replacement by proliferating tissue with polymorphonuclear cells Haematoxylin and eosin (H&E), magnification 10 ×

**Figure 2:**
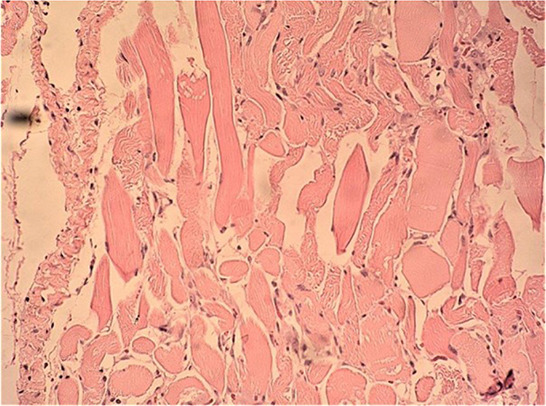
Swelling of muscle fibres with loss of their internal structure, oedema of the endomysium with focal polymorphonuclear cell infiltration Haematoxylin and eosin (H&E), magnification 20 ×

**Figure 3:**
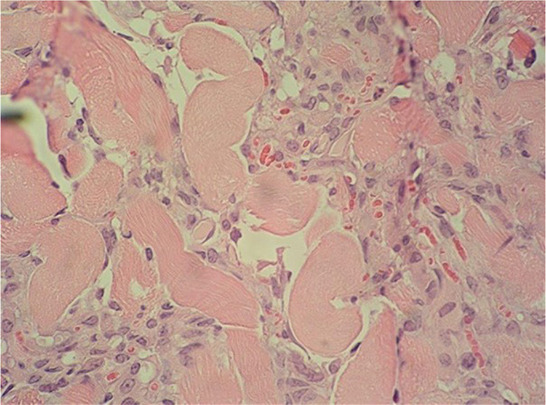
Focal granulation to necrosis of the muscle fibres with a mild cellular reaction in their surroundings Haematoxylin and eosin (H&E), magnification 40 ×

In the skeletal muscles, myocytes were pale and deformed showing granulation of the cytoplasm and partial loss of transverse striation. Prominent diffuse focal degeneration and destruction of the muscle fibres with their replacement by proliferating fibrous tissue infiltrated by histiocytes and mononuclear cells were apparent. The vessels were unchanged, the endomysium was slightly oedematous. A small number of sarcocysts was detected in the muscles (Figures 1–3).

The histopathological examination of the kidney revealed the vacuolisation and granulation of the tubular epithelium of the cortex, eosinophilic hyaline droplets in the tubule lumen, and enlarged glomeruli with hyaline droplets in Bowman’s capsule.

A rare focal microvesicular vacuolation of hepatocytes occurred in the liver. Extracellular oedema in both white and grey matter without inflammatory cell infiltration was found in the cerebrum.

The virological examination of the content of the digestive system performed in the animal that died on the 6^th^ day was negative. The bacteriological examination of the liver and kidney in the same animal revealed the presence of isolated saprophytic bacteria only. The following bacteria were detected in the intestine: *Proteus vulgaris* ++, *Bacillus circulans* ++, *Bacillus* sp. +, *Acinetobacter lwofii* +, *Streptococcus mutans* +. The examination for *Salmonella* sp. and *Clostridium perfringens* was negative.

The examination of the intestinal content using flotation was negative. The rumen fluid pH was 6.34. No presence of funnels was detected.

The results of the biochemical examination are shown in Table 1. Blood taken six days after the initiation of the contaminated feed consumption from a fallow deer with clinical signs of poisoning had an elevated urea level with a normal creatinine level and extremely elevated CK activity, hypermagnesemia, marked hyponatremia, marked hypochloraemia and extreme hyperkalaemia. The biochemical analyses of blood sampled from a deer on the 12^th^ day showed elevated concentrations of urea, magnesium, and potassium, an increase in the activity of CK and ALT, and a decrease in the sodium and chloride concentrations. The creatinine concentration remained within the normal range.

**Table 1:** Results of the biochemical analysis at 6 and 12-days of exposure in comparison to the physiological rank in fallow deer by other authors

Parameter	Adult females, 6-days exposure	Juvenile, 11-days exposure	Adult females, different age (Chapman et al. 1980) range	Non-pregnant and pregnant females (Poljicak-Milas et al. 2009) mean ± SD	Farmed female (Vengust et al. 2002) mean ± SD	Juvenile animals 20–21 months (Tajchman et al. 2023) range
Total protein (g/l)	74.3 76.0	57.4	54.0–74.0	70.6 ± 9.3 70.6 ± 9.3	61.1 ± 8.1	46.0–79.0
Albumin (g/l)	31.6 34.0	27.7	27.0–49.0	36.3 ± 3.7 43.3 ± 3.4	37.7 ± 8.9	30.0–50.0
Globulins (g/l)	42.7	29.7	–	–	–	–
AST (μkat/l)	0.5	0.3	0.5–1.4	–	3.0 ± 2.0	–
ALT (μkat/l)	**> 17.000 ↑**	–	–	0.46 ± 0.14 0.61 ± 0.08	0.81 ± 0.22	–
ALP (μkat/l)	0.57	–	0.03–0.61	–	–	–
GMT (GGT) (μkat/l)	0.92 0.817	**2.39 ↑**	–	–	0.59 ± 0.30	–
Urea (mmol/l)	**12.69** **16.00 ↑**	**30.33 ↑**	–	6.4 ± 3.4 11.1 ± 0.6	5.7 ±2.3	–
Creatinine (μmol/l)	102.0 **> 5 000 ↑**	112	–	131.3 ± 3.4 156.0 ± 14.7	148.7 ± 70.7	99.0–129.1
CK (μkat/l)	**> 100 ↑**	**> 100 ↑**	0.5–2.6	–	–	–
Bilirubin total (μmol/l)	– 11.0	–	–	–	–	–
Glucose (mmol/l)	– **15.6 ↑**	–	–	6.1 ± 2.3 4.7 ± 0.3	5.0 ± 4.0	4.4–6.8
Cholesterol (mmol/l)	– 1.75	–	–	1.91 ± 0.19 3.14 ± 0.53	–	1.37–2.38
Ca (mmol/l)	1.5 1.3	2.3	1.8–2.8	–	2.6 ± 0.3	–
P (mmol/l)	**8.2** **> 5.2 ↑**	2.4	1.4–2.7	–	3.9 ± 1.1	–
Mg (mmol/l)	1.6 1.6	4.6	–	–	1.2 ± 0.2	–
Na (mmol/l)	**103.7 ↓**	134.9	–	–	147.4 ± 4.8	–
K (mmol/l)	**34.4 ↑**	6.3	–	–	12.1 ± 3.3	–
Chlorides (mmol/l)	82.9	91.0	–	–	107.0 ± 3.2	–
Cu (μmol/l)	– 13.6	–	–	–	16.0 ± 3.2	–
Zn (μmol/l)	– 7.8	–	–	–	–	–
Se (μg/l)	–	96.8	–	–	–	–

ALT = alanine transaminase; AST = aspartat aminotransferase; Ca = calcium; CK= creatin kinase; GMT = gama-glutamyltransferase; K = potassium; Mg = magnesium; Na = sodium; P = phosphorus; Se = selenium; Zn = zinc

The haematological examination revealed pronounced panleukopenia caused primarily by lymphocytopenia (Table 2).

**Table 2:** Results of the haematological analysis at 12-days of exposure in comparison to the physiological rank of fallow deer by other authors

Parameter	Juvenile	Adult female, farmed (Agar and Godwin 1992) mean ± SEM	Non-pregnant and pregnant females (Poljicak-Milas et al. 2009) mean ± SD	Juvenile animals (Kovac et al. 1997) mean ± SD	Male and female, 1–2 years (Chapman et al. 1982) range	Juvenile animals 20–21 months (Tajchman et al. 2023) range
RBC (T/l)	12.1	9.9 ± 0.1	11.6 ± 0.9 8.8 ± 0.7	11.0 ± 2.1	11.9–14.4	11.4–13.6
Hb (g/l)	131.0	172.0 ± 3.0	152.0 ± 12.1 122.8 ± 8.9	131.2 ± 23.5	159.0–206.0	172.0–192.0
PCV (l/l)	**0.36 ↓**	0.47 ± 0.01	0.54 ± 0.01 0.368 ± 0.004	0.48 ± 0.05	0.43–0.53	0.44–0.50
Thrombocytes (G/l)	107.0	–	–	–	–	92.0–383.0
MCV (fL)	**29.5 ↓**	47.5 ± 0.6	47.0 ± 4.8 41.2 ± 1.7	–	34.0–37.0	35.9–38.1
MCH (pg)	**10.8 ↓**	17.4 ± 0.2	13.2 ± 1.0 13.8 ± 1.1	–	12.0–14.0	13.8–14.5
MCHC (g/l)	367	366 ± 8.0	283 ± 17 312 ± 7.0	–	360–390	371–396
WBC (G/l)	**1.38 ↓**	–	3.44 ± 0.80 4.95 ± 0.91	3.98 ± 1.48	1.87–4.90	3.47–5.33
Neutrophils segmented (G/l)	0.88 (63.6%)	–	2.41 ± 0.09 (76–81%) 2.41 ± 0.08 (37–78%)	19–50%	0–0.6 (band and segm.)	–
Neutrophils band (G/l)	0	–	–	0–28%	–	–
Lymphocytes (G/l)	**0.38 ↓** (27.2%)	–	0.83 ± 0.18 (19–42%) 1.83 ± 0.76 (16–58%)	38–64%	0.66–1.52	–
Monocytes (G/l)	0.13 (9.1%)	–	–	0–4%	0	–
Eosinophiles (G/l)	0	–	0.02 ± 0.02 (0–2%) 0.64 ± 0.53 (2–30%)	0–18%	0–0.59	–
Basophiles (G/l)	0	–	–	0	0	–

Hb = haemoglobin concentration; MCH = mean corpuscular haemoglobin; MCHC = mean corpuscular haemoglobin concentration; MCV = mean corpuscular volume; PCV = packed cell volume; RBC = red blood cells count; WBC = white blood cells count

The concentration of ivermectin measured in the blood 6 days after the exposure was 12.09 μg/kg. Ivermectin was not detected in the blood on the 12^th^ day after the medicated feed consumption.

The medicated feed contained ivermectin in the concentration of 13.1 mg/kg and sodium salinomycinate in the concentration of 252.6 mg/kg feed.

## DISCUSSION

Two active substances detected in the medicated feed used in the affected deer farm, ivermectin and salinomycin, have different therapeutic uses and therefore have different mechanisms of action. Ivermectin, belonging to avermectins, is, together with milbemycins, often referred to as being a macrocyclic lactone. Macrocyclic lactones are widely used in veterinary medicine for the treatment of gastrointestinal nematode infections and ectoparasite infestations. The molecular targets of the avermectin anthelmintics are the glutamate-gated chloride channels (GluCl). Avermectins also interact with other invertebrate and vertebrate ligand-gated chloride channels (Wolstenholme and Rogers 2005). The closest homologues of GluCl in vertebrates are probably the glycine-gated chloride channels (Vassilatis et al. 1997). The selective toxicity of avermectins for parasites over their vertebrate hosts is not caused by the higher affinity at the GluCl channels over other channels, but mainly by the fact that avermectins are good substrates for P-glycoproteins. These pumps in vertebrates most likely reduce the toxicity of avermectins for the host versus the parasite due to their removal from the central nervous system (CNS) by non-specific pumps of the blood-brain barrier (Nobmann et al. 2001).

The detected blood level of ivermectin did not exceed the levels demonstrated when administered in a therapeutic dose in reindeer. According to an European Agency for the Evaluation of Medicinal Products report (EMEA 1998), the maximum plasma concentration of ivermectin in reindeer after a therapeutic subcutaneous dose of 0.2 mg/kg b.w. is reached approximately 28 h after the treatment with a peak plasma concentration of 15.3 μg/l. The plasma half-life of ivermectin is 4 days (EMEA 1998). The concentration of ivermectin in the serum (12.09 μg/kg) of the fallow deer 4 days after the feeding of the medicated feed therefore corresponds to the therapeutical concentration. The concentration of ivermectin in the plasma below the limit of quantification (< 5.00 μg/kg) in the fallow deer 12 days after exposure reflects the replacement of the feed four days after. The concentration of ivermectin of 13.1 mg/kg as analysed in the medicated feed also corresponds to the producer’s label concentration of 15 mg/kg. The therapeutical dose was, therefore, not exceeded, and we do not anticipate the toxic effect of ivermectin in this case.

Another active substance contaminating feed in this deer farm, salinomycin sodium, belonging to carboxylic ionophores, is authorised in the EU as a coccidiostatic feed additive in poultry and classified as an additive substance for medicated feed under Regulation (EC) No. 4/2019. It differs from ivermectin, which is a veterinary medical product governed by Regulation (EC) No. 6/2019. A high concentration of salinomycin sodium in the medicated feed (252.6 mg/kg) proved to cause the accidental intoxication of fallow deer by this substance.

Clinical symptoms found in our case, i.e., anorexia, dyspnoea and ataxia without signs of convulsions, were similar to those described by Omidi et al. (2010) in calves. Calves exposed to a salinomycin concentrate 70 g/kg were anorexic, depressed, weak, ataxic, and had severe dyspnoea.

The pathological findings in our case are also typical for ionophore intoxication in ruminants, although no case report of ionophore intoxication in fallow deer has been filed.

The results of the histopathological examination on the eleventh day after exposure in our case revealed the pronounced focal acute cardiomyopathy of the heart and massive subacute myopathy of skeletal muscles which are common findings in cases of poisoning with ionophore antibiotics (Galitzer et al. 1986). The vacuolar degeneration of the cardiac and skeletal muscles in calves who died within 48-hours after poisoning with salinomycin in the feed in a concentration of 420–810 mg/kg were described by Huyben et al. (2001). The severe haemorrhage in the epicardium, foci of degenerate and necrotic cardiac muscle cells in the ventricular myocardium, extensive subepicardial haemorrhages, oedema and haemorrhages between the cardiomyocyte bundles, extensive myocardial degeneration and necrosis (cardiomyolysis) were reported in calves and sheep who died a short time after exposure to high concentrations of salinomycin (Omidi et al. 2010; Ashrafihelan et al. 2014). The non-specific findings in the histopathological examination that we found in the myocard of fallow deer four days after exposure were probably caused by a lower salinomycin concentration in comparison to the previous studies.

The fallow deer intoxication with salinomycin is also confirmed by the results of the biochemical examination. Salinomycin forms lipid soluble dynamically reversible complexes with cations and facilitates a specific ionic transport across biologic membranes. The ionophoric activity may alter normal concentration gradients resulting in a cellular ion imbalance, pH change, lipid peroxidation, and disruption of plasma membranes (Novilla 2018). Salinomycin preferentially binds K^+^ over Na^+^. The initial efflux of potassium outside the cells may result in intracellular acidosis due to the H^+^ influx. This increase in the potassium concentration was apparent in the serum of fallow deer after 6 days of exposure followed by a decline after 11 days. The decrease in the sodium concentration in the serum after 6 days of exposure could be explained by an influx of Na^+^ into the cells counterbalanced by an efflux of K^+^. The most significantly affected parameters above all the biochemical indices in the 6 days of exposure were an increase in the creatinine, as well as the creatine kinase and ALP activities. The increase in these enzymes was likely caused by the cardiac and skeletal muscle cell disruption. The high concentration of creatinine in the serum, which is excreted by glomerular filtration in the kidneys, could be a reason for kidney failure and the increase in urea and total phosphorus concentrations on the same sampling day. The kidney failure also indicates high concentrations of urea in the juvenile fallow deer sampled 11 days after the exposure. Concerning the serum enzymes, the significant findings in the same animal showed high activities of creatine kinase and GMT.

Haematologic parameters are not significantly affected by ionophores. No signs of toxicity detectable by the haematological parameters were observed at dietary monensin concentrations of up to 110 mg/kg for beef cattle, 120 mg/kg for calves and 500 mg/kg for pigs (EFSA 2006; EFSA 2008). The post-dosing occurrence of leukocytosis after an oral dose mycelial monensin in cattle was described by Van Vleet et al. (1983). Contrary to these reports, we found leukopenia, lymphocytopenia, a lower PCV, volume of RBC and related MCH in fallow deer 6 days after the exposure. The hyperaemia of organs and haemorrhagic content in the intestine found at the gross necropsy explain the decrease in the number of blood cells in terms of the lymphocyte numbers and in terms of the PCV and RBC volume.

The results of the acute toxicity studies with ionophores showed that the most sensitive animal is the horse, but dose-depending lethal effects were described in many domestic animals such as cattle, sheep, pigs, turkeys, dogs, and cats (e.g., Wilson 1980; Potter et al. 1986; van der Linde-Sipman et al. 1999; Huyben et al. 2001; Kart and Bilgili 2008; Omidi et al. 2010; Novilla 2018). The acute toxicity of ionophores in cervids has not been recorded and this case of accidental salinomycin poisoning showed the higher sensitivity of farmed fallow deer in comparison to red deer. During the *ad libitum* feeding of medicated feed contaminated by salinomycin in the concentration of 252.6 mg/kg for four days, 71% of the farmed fallow deer died while none of the eight red deer died.

We have also taken the possible interaction between the action of ivermectin and salinomycin into consideration. Biotransformation of ionophores via *O*-demethylation is mediated by the cytochrome P450 (CYP 450) enzyme family. Depending on the total amount of CYP450 involved in the biotransformation and the rate of *O*-demethylation, the ionophore toxicity varies considerably among animal species along with the ionophore type (Nebbia et al. 2001; Novilla 2018). Many studies reported the interaction of ionophores with various antibiotics in chickens, pigs and turkeys as possible consequences of altered metabolisation by CYP 450 enzymes (e.g., Wang et al. 2013 – florfenicol; Nebbia et al. 1999 – tiamulin; Oehme and Pickrell 1999 – furazolidone; Ekinci et al. 2023). The interaction between ionophores and antiparasitic drugs has not yet been described, however, the effect of ivermectin on cytochromes P450 activities in fallow deer was investigated by Skalova et al. (2001). Repeated doses of 0.5 mg of ivermectin per kilogram of body weight caused a slight increase in the BROD (benzyloxyresorufin *O*-dealkylase) activity, but no elevation of the MROD (7-methoxyresorufin *O*-dealkylase), EROD (7-ethoxyresorufin *O*-dealkylase), and PROD (7-pentoxyresofufin *O*-dealkylase) activities. These findings implicate that the therapeutical dose of 0.2 mg/kg body weight (b.w.) probably does not affect the toxicity of salinomycin and the reason for the different sensitivity of red deer and fallow deer to salinomycin could be due to differences in its metabolisation.

According to the results of the pathological and histopathological examinations of the myocardium and skeletal muscles, intoxications with selenium, hypoglycin A and capture myopathy might be considered as possible causes of the mortalities. The mild myocardial sarcocystosis found in the necropsy of both animals is a common finding and we do not expect this to have any effect on the mortality or any other analysed parameters. Intoxication with selenium is unlikely because the level of selenium in the serum (96.8 μg/l) did not exceed the levels detected in other ruminant species. For example, according to Weiss et al. (1990), a serum Se concentration of 80 μg/l is considered necessary for the maximal immune response against mastitis pathogens in dairy cows. We can also exclude selenium intoxication because, in the case of selenium, changes are typically found mainly in the myocardium, unlike salinomycin, where there are changes both in the myocardium and skeletal muscles, as in our case.

From the anamnestic point of view, we consider intoxication by hypoglycin A contained in maple trees unlikely and we excluded capture myopathy as well. There were no fragments of maple leaves and achenes in the gastrointestinal content. Moreover, we demonstrated more serious changes in the myocardium, whereas, in cases of intoxications with hypoglycin A, changes in skeletal muscles prevail (Bochnia et al. 2021).

Capture myopathy – a non-infectious disorder of wild and domestic animals, in which muscle damage is caused by extreme exertion, combat, or stress often occurs as a result of chemical immobilisation, capture, or transport, but may also be the result of other stress factors. Capture myopathy due to a chemical immobilisation in spotted deer which was characterised by a loss of striations and mild granular degeneration in the heart and distorted fibres and a loss of striation in skeletal muscles was described by Ashraf et al. (2019).

Excluding infections and other adverse reasons, we can conclude that the main cause of death of the fallow deer in the described case was the severe damage to the skeletal muscles and myocardium (myopathy/myocardiopathy) with a subsequent circulatory insufficiency due to intoxication with a high concentration of salinomycin sodium from an accidentally contaminated medicated feed. The pathological and biochemical findings correspond to the toxic effect of this substance.
